# Prospective associations between vitamin D and depression in middle-aged adults: findings from the UK Biobank cohort

**DOI:** 10.1017/S0033291720003657

**Published:** 2022-07

**Authors:** Amy Ronaldson, Jorge Arias de la Torre, Fiona Gaughran, Ioannis Bakolis, Stephani L. Hatch, Matthew Hotopf, Alexandru Dregan

**Affiliations:** 1 Institute of Psychiatry, Psychology and Neuroscience (IoPPN), King's College London, London, UK; 2 CIBER Epidemiology and Public Health (CIBERESP), Madrid, Spain; 3 Institute of Biomedicine (IBIOMED), University of Leon, Leon, Spain; 4 South London and Maudsley NHS Foundation Trust, London, UK; 5 ESRC Centre for Society and Mental Health, King's College London, London, UK

**Keywords:** Depression, middle-age, PHQ-9, vitamin D

## Abstract

**Background:**

A possible role of vitamin D in the pathophysiology of depression is currently speculative, with more rigorous research needed to assess this association in large adult populations. The current study assesses prospective associations between vitamin D status and depression in middle-aged adults enrolled in the UK Biobank.

**Methods:**

We assessed prospective associations between vitamin D status at the baseline assessment (2006–2010) and depression measured at the follow-up assessment (2016) in 139 128 adults registered with the UK Biobank.

**Results:**

Amongst participants with no depression at baseline (*n* = 127 244), logistic regression revealed that those with vitamin D insufficiency [adjusted odds ratio (aOR) = 1.14, 95% confidence interval (CI) = 1.07–1.22] and those with vitamin D deficiency (aOR = 1.24, 95% CI 1.13–1.36) were more likely to develop new-onset depression at follow-up compared with those with optimal vitamin D levels after adjustment for a wide range of relevant covariates. Similar prospective associations were reported for those with depression at baseline (*n* = 11 884) (insufficiency: aOR = 1.11, 95% CI 1.00–1.23; deficiency: aOR = 1.30, 95% CI 1.13–1.50).

**Conclusions:**

The prospective associations found between vitamin D status and depression suggest that both vitamin D deficiency and insufficiency might be risk factors for the development of new-onset depression in middle-aged adults. Moreover, vitamin D deficiency (and to a lesser extent insufficiency) might be a predictor of sustained depressive symptoms in those who are already depressed. Vitamin D deficiency and insufficiency is very common, meaning that these findings have significant implications for public health.

## Introduction

Vitamin D deficiency is now recognized as a major global health problem (Holick & Chen, 2008). In the UK, rates of vitamin D deficiency and insufficiency are considerable which might be explained by lack of sunlight exposure, diet and increases in sedentary indoor lifestyles (Davies & Shaw, 2011). Vitamin D is considered essential for bone health and exerts effects on blood pressure, glycaemic control and immune function (Parker, Brotchie, & Graham, 2017). Being deficient in vitamin D has been associated with the development of cancer, infectious diseases and autoimmune diseases (Holick & Chen, 2008). Vitamin D is also thought to impact upon the expression of certain neurotransmitters and vitamin D receptors are present on several brain areas known to be implicated in depression (Eyles, Burne, & McGrath, 2013). Therefore, in recent years, vitamin D has emerged as a possible factor related to the development of depression.

To date, epidemiological studies have provided evidence for cross-sectional associations between vitamin D levels and depression (Anglin, Samaan, Walter, & McDonald, 2013; Parker et al., 2017). Three meta-analyses of longitudinal studies where vitamin D status was used to predict later depression have been carried out in older adults (Anglin et al., 2013; Ju, Lee, & Jeong, 2013; Li et al., 2019). Results of these meta-analyses support the notion that lower vitamin D levels play a role in the development of depression. However, the largest meta-analysis to date had a sample of just over 43 000, and meta-analyses are prone to reporting bias in favour of positive results. The UK Biobank is a large, long-term biobank study based in the UK which provides an opportunity for the analysis of associations between vitamin D and depression in a sample which is more than three times larger than the world literature. Moreover, the majority of evidence to date comes from studies carried out in older adults over 65 years of age. Ageing is known to bring about a reduction in vitamin D production (Gallagher, 2013) and risk factors for depression are also known to change across the lifespan (Muñoz, Beardslee, & Leykin, 2012). Examining associations between vitamin D status and depression among middle-aged adults might yield different results.

We propose to examine prospective associations between vitamin D status and new-onset depression using large-scale data from the UK Biobank. Given the high levels of vitamin D deficiency and insufficiency in the UK, examining prospective associations between vitamin D status and depressive symptoms could have significant public health implications.

## Method

### Study design and participants

Data were collected from UK Biobank (*n* = 502 640) participants aged between 40 and 69 years from 22 different assessment centres across England, Scotland and Wales between 2006 and 2010 (Sudlow et al., 2015). Participants had to be registered with a general practitioner (GP) and live within 25 miles of an assessment centre to take part. All participants gave informed consent. Detailed accounts of sociodemographic, lifestyle and medical information were gathered from all participants recruited to the study using a touchscreen questionnaire during the baseline assessment. Participants also provided information about medical diagnoses in a computer-assisted personal interview administered by trained interviewers. Blood samples were collected from participants during the baseline assessment in order to measure a number of biological factors including vitamin D levels. In 2016, an online questionnaire was completed by 157 366 participants to collect follow-up information about mental health (Davis et al., 2020). The UK Biobank has ethical approval from the NHS National Research Ethics Service (16/NW/0274).

In the current observational cohort study, the sample was selected based on participants who provided both a vitamin D measurement at the baseline assessment and who completed the follow-up online mental health questionnaire. We conducted two separate analyses. First, cross-sectional associations between vitamin D and depression were examined at baseline in the entire sample. Secondly, prospective associations between vitamin D levels at baseline and depression at follow-up stratified by depression status at baseline were examined in order to investigate (a) incident depression in people with no depression at baseline, and (b) chronic sustained depressive symptomatology in those with depression at baseline.

### Vitamin D

Serum levels of 25-hydroxyvitamin D (25OHD) were measured using a chemiluminescent immunoassay (DiaSorin Liaison XL, DiaSorin Ltd., UK). The assay for 25OHD had an analytical sensitivity (lower detection limit) of 10 nmol/L. The detection limit represents the lowest measurable analyte level that can be distinguished from zero. The coefficient of variation ranged from 5.04% to 6.14%. We used recommended thresholds from UK Scientific Advisory Committee on Nutrition (SACN) to identify participants who had sufficient levels of vitamin D (>50 nmol/L), insufficient levels of vitamin D (20–50 nmol/L) and those who were deficient in vitamin D (<20 nmol/L) (SACN, 2016). This classification was used for the present study.

### Depression

This study used multiple sources to determine depression at baseline: self-report, the Patient Health Questionnaire (PHQ)-2 (Kroenke, Spitzer, & Williams, 2003) and linked hospital admission records. A detailed description of how depression at baseline was measured in the current study is provided in previous work assessing depression using UK Biobank data (Dregan et al., 2020).

The PHQ-9 was used to assess depression at follow-up (Kroenke, Spitzer, & Williams, 2001). The PHQ-9 is a nine-item questionnaire which scores each of the nine DSM-IV criteria for depression as ‘0’ (‘not at all’) to ‘3’ (‘nearly every day’). Scores range from 0 to 27. A score of 10 or higher is indicative of depression (Mitchell, Yadegarfar, Gill, & Stubbs, 2016), and this was used to create a binary depression outcome variable. The PHQ-9 has been shown to be a valid and reliable diagnostic tool for possible depression in a variety of populations (Levis, Benedetti, & Thombs, 2019; Moriarty, Gilbody, McMillan, & Manea, 2015). A direct comparison of the diagnostic ability of the PHQ-2 and the PHQ-9 shows that they perform similarly (Löwe et al., 2004).

### Covariates

Several covariates were selected and were informed by previous literature. Sociodemographic variables included age, sex, ethnicity (white/non-white), and socioeconomic status (SES). SES was measured using the Townsend Social Deprivation Index (Townsend, 1987). Height and weight were collected during the baseline assessment and used to derive body mass index (BMI) using the standard formula (kg/m^2^). Whether or not participants reported being a current or past smoker (yes/no) and the number of units of alcohol consumed per week were also included. Physical activity was assessed with a modified version of the International Physical Activity Questionnaire that recorded total physical activity (e.g. mild, moderate, vigorous) performed over the previous 7 days. Accordingly, participants were classified into four mutually exclusive categories: none, low [<600 metabolic equivalent (MET) minutes/week], moderate (600 to <3000 MET) or high (3000+ MET). As the presence of chronic physical conditions is known to predict new-onset depression (Read, Sharpe, Modini, & Dear, 2017), we also adjusted for the number of physical health conditions each participant reported having at the baseline assessment. The physical health conditions reported by participants were organized into 37 long-term conditions based on previously published literature on multimorbidity (Barnett et al., 2012; Jani et al., 2019). The season in which the blood sample was taken was also adjusted for as this can directly impact vitamin D levels (Lagunova, Porojnicu, Lindberg, Hexeberg, & Moan, 2009).

### Statistical analysis

Variables are summarized as means and standard deviations for continuous variables and frequencies for discrete variables. Intergroup comparisons between different vitamin D status groups were performed using analysis of variance and χ^2^ tests. Unadjusted, age/sex-adjusted and fully adjusted potential associations between vitamin D and depression were explored with the use of logistic and linear regression models. Prospective associations were examined separately in those with depression and those without depression at baseline.

We adjusted our analysis for *a priori* confounders such as age, sex, ethnicity, SES, BMI, smoking status, weekly alcohol intake, physical activity, number of self-reported physical long-term conditions and season in which the blood sample was taken.

Missing data for study variables ranged from 0% (SES) to 25% (alcohol intake). Multiple imputation using chained equations with 10 imputations was performed to optimize the validity of the study findings.

Results for these models are presented as adjusted odds ratios (aOR) with 95% confidence intervals (CI). All analyses were conducted using STATA 15.1 (Stata Corp LLP, College Station, TX, USA).

### Sensitivity analyses

Five sensitivity analyses were carried out. Firstly, we sought to examine associations between vitamin D and depression with alcohol intake omitted from the regression models as the missing data that were imputed exceeded the recommended threshold of 15%.

The second sensitivity analysis related to vitamin D status thresholds. The current study uses the UK SACN thresholds to determine vitamin D status (deficiency: <20 nmol/L, insufficiency: 20–50 nmol/L, sufficiency: >50 nmol/L). However, the US Institute of Medicine (IOM) has recommended that vitamin D thresholds should be defined as follows: <30 nmol/L denotes deficiency; 30–50 nmol/L denotes insufficiency; >50 nmol/L denotes sufficient vitamin D levels [Institute of Medicine (US) Committee to Review Dietary Reference Intakes for Vitamin D and Calcium, 2011]. We reran the analyses (linear regression) using the US IOM vitamin D thresholds for comparative purposes.

The third sensitivity analysis was performed to assess the impact of vitamin D status on continuous PHQ-9 scores using linear regression.

Fourth, we sought to assess associations between vitamin D status at baseline and depression at follow-up in the entire sample, including baseline depression in the model as a covariate.

Finally, the robustness of the fully-adjusted cross-sectional and prospective results to unmeasured confounding was assessed using the E-value methodology (VanderWeele & Ding, 2017).

## Results

### Sample characteristics

Analysis was carried out in participants who had Vitamin D levels measured at baseline and completed the follow-up mental health questionnaire (*n* = 139 128). Sample characteristics for participants are described in Table 1. The majority of the sample were either insufficient (42.2%) or deficient (11.7%) in vitamin D at baseline, with 46.1% having sufficient levels. Participants with deficient levels of vitamin D were more likely to be female (56.7%) compared to those with insufficient (55.6%) and sufficient (55.6%) levels. Those with insufficiency (55.46 ± 7.76 years) and deficiency (53.95 ± 7.71 years) were younger than those with sufficient levels (56.63 ± 7.67 years) of vitamin D. They were also more likely to have higher levels of deprivation (deficient: −1.04 ± 3.14; insufficient: −1.60 ± 2.86; sufficient: −1.99 ± 2.67), and were more likely to be of non-white ethnicity (deficient: 8.4%; insufficient: 3.2%; sufficient: 1.1%). People with sufficient levels of vitamin D were more likely to engage in high levels of physical activity (45.6%) compared to those with insufficiency (36.0%) and deficiency (29.2%).

**Table 1.** Sample characteristics according to vitamin D status (SACN) at baseline

	All participants (*n* = 139 128)	Sufficient (*n* = 64 147, 46.1%)	Insufficient (*n* = 58 724, 42.2%)	Deficient (*n* = 16 257, 11.7%)	*p* value
	Mean ± s.d. or *N*(%)	Mean ± s.d. or *N*(%)	Mean ± s.d. or *N*(%)	Mean ± s.d. or *N*(%)	
Age	55.83 ± 7.76	56.63 ± 7.67	55.46 ± 7.76	53.95 ± 7.71	<0.0001
Female	77 535 (55.7)	35 644 (55.6)	32 672 (55.6)	9219 (56.7)	0.027
BMI (kg/m^2^)	26.75 ± 4.55	26.07 ± 3.99	27.19 ± 4.73	27.87 ± 5.48	<0.0001
Non-white ethnicity	3959 (2.9)	714 (1.1)	1885 (3.2)	1360 (8.4)	<0.001
Townsend deprivation Index	−1.72 ± 2.83	−1.99 ± 2.67	−1.60 ± 2.86	−1.04 ± 3.14	<0.0001
Smoking status					0.001
Past/current smoker	59 220 (42.6)	27 649 (43.1)	24 681 (42.0)	6890 (42.4)	
Never smoked	79 908 (57.4)	36 498 (56.9)	34 043 (58.0)	9367 (57.6)	
Alcohol intake (*units per week*)	21.08 ± 16.82	21.14 ± 16.54	20.85 ± 16.80	21.62 ± 17.98	<0.0001
Physical activity					<0.0001
None	1537 (1.1)	506 (0.8)	672 (1.1)	359 (2.2)	
Low	24 102 (17.3)	9035 (14.1)	11 190 (19.1)	3877 (23.9)	
Moderate	58 351 (41.9)	25 361 (39.5)	25 718 (43.8)	7272 (44.7)	
High	55 138 (39.6)	29 245 (45.6)	21 144 (36.0)	4749 (29.2)	
Vitamin D (*nmol/L*)	49.47 ± 20.75	67.61 ± 14.35	37.94 ± 7.10	19.55 ± 3.75	<0.0001
Vitamin D status (IOM)					<0.001
Sufficient	64 147 (46.1)	64 147 (100.0)	–	–	
Insufficient	48 609 (34.9)	–	48 609 (82.8)	–	
Deficient	26 372 (19.0)	–	10 115 (17.2)	16 257 (100.0)	
Baseline depression					<0.001
Depression	11 884 (8.5)	4730 (7.4)	5298 (9.0)	1856 (11.4)	
No depression	127 244 (91.5)	59 417 (92.6)	53 426 (91.0)	14 401 (88.6)	
PHQ-9 follow-up	2.76 ± 3.68	2.48 ± 3.41	2.86 ± 3.77	3.41 ± 4.25	<0.0001
PHQ-9 depression					<0.001
Depression	7937 (5.7)	2950 (4.6)	3617 (6.2)	1370 (8.4)	
No depression	131 191 (94.3)	61 197 (95.4)	55 107 (93.8)	14 887 (91.6)	
Physical disease count	0.99 ± 1.08	0.98 ± 1.07	0.98 ± 1.08	1.02 ± 1.14	<0.0001
Season of blood sampling					<0.001
Spring	28 238 (20.3)	12 834 (20.0)	19 314 (32.9)	7233 (44.4)	
Summer	39 381 (28.3)	23 837 (37.2)	12 276 (20.9)	1091 (6.7)	
Autumn	37 204 (26.7)	19 121 (29.8)	13 169 (22.4)	2015 (12.4)	
Winter	34 305 (24.7)	8355 (13.0)	13 965 (23.8)	5918 (36.4)	

BMI, body mass index; IOM, US Institute of Medicine; nmol, nanomole; PHQ, Patient Health Questionnaire; SACN, Scientific Advisory Committee on Nutrition; s.d., standard deviation

In the overall sample, 8.5% of participants reported depression at baseline and this differed significantly across vitamin D levels in a dose–response manner (deficient: 11.4%; insufficient: 9.0%; sufficient: 7.4%). Those with depression at baseline were younger (depression: 54.1 ± 7.5 years; no depression: 56.0 ± 7.8 years), and more likely to be male (64.7% with depression *v.* 54.9% no depression). They also had higher deprivation (depression: −1.2 ± 3.1; no depression: −1.8 ± 2.8), and were more likely to be of non-white ethnicity (depression: 3.9%; no depression: 2.8%). People with depression had higher BMI (depression: 27.7 ± 5.4; no depression: 26.7 ± 4.5), were more likely to be past or current smokers (depression: 46.4%; no depression: 42.2%) and drank more units of alcohol per week (depression: 22.6 ± 18.4; no depression: 20.9 ± 16.7). They also had more physical long-term conditions (1.3 ± 1.3) than those without depression (1.0 ± 1.1).

### Cross-sectional associations between vitamin D and depression at baseline

After adjusting for all *a priori* confounders, the findings indicated a dose–response association between vitamin D levels and depression at baseline (Table 2). Specifically, there was strong evidence for an increased risk of depression among participants with insufficient levels of vitamin D (aOR: 1.16; 95% CI 1.11–1.21) and among those with deficient levels of vitamin D (aOR: 1.34; 95% CI 1.25–1.42) compared to participants with optimal vitamin D levels. Our findings were consistent when vitamin D status was entered into the fully-adjusted model as a continuous variable. There was evidence for a negative association with depression at baseline for every unit increase in vitamin D (nmol/L) (aOR = 0.995, 95% CI 0.994–0.996) (Table 2).

**Table 2.** Cross-sectional associations between vitamin D (SACN thresholds) and depression caseness at baseline

	Unadjusted		Age and sex adjusted		Fully adjusted^a^	
	OR (95% CI)	*p* value	OR (95% CI)	*p* value	OR (95% CI)	*p* value
Vitamin D	0.992 (0.991–0.993)^b^	<0.001	0.993 (0.992–0.994)^b^	<0.001	0.995 (0.994–0.996)^b^	<0.001
Sufficient	Reference		Reference		Reference	
Insufficient	1.25 (1.20–1.30)	<0.001	1.21 (1.16–1.26)	<0.001	1.16 (1.11–1.21)	<0.001
Deficient	1.62 (1.53–1.71)	<0.001	1.50 (1.42–1.59)	<0.001	1.34 (1.25–1.42)	<0.001

a Covariates: age, sex, deprivation, ethnicity, BMI, smoking, weekly alcohol intake, physical activity, physical disease count and season of blood sampling.

b Odds ratio for vitamin D (nmol/L) treated as a continuous value.

### Prospective associations between vitamin D and depression at follow-up

Prospective associations between vitamin D and depression at follow-up are presented in Table 3. Among people who did not have depression at baseline, the fully-adjusted model revealed that both vitamin D insufficiency (aOR = 1.13, 95% CI 1.06–1.20) and deficiency (aOR = 1.22, 95% CI 1.11–1.34) were associated with an increased likelihood of developing new depression at follow-up. When vitamin D was entered into the fully-adjusted model as a continuous variable, the results indicated that for every unit (nmol/L) increase in vitamin D, the risk of new depression declined modestly (aOR = 0.996, 95% CI 0.994–0.997).

**Table 3.** Prospective associations between vitamin D (SACN thresholds) and depression at follow-up (PHQ-9)

Depression at baseline assessment (*n* = 11 884)						
	Unadjusted		Age and sex adjusted		Fully adjusted^a^	
	OR (95% CI)	*p* value	OR (95% CI)	*p* value	OR (95% CI)	*p* value
Vitamin D	0.991 (0.988–0.993)^b^	<0.001	0.992 (0.990–0.994)^b^	<0.001	0.996 (0.994–0.999)^b^	0.003
Sufficient	Reference		Reference		Reference	
Insufficient	1.30 (1.18–1.43)	<0.001	1.24 (1.12–1.36)	<0.001	1.11 (1.00–1.23)	0.042
Deficient	1.80 (1.60–2.04)	<0.001	1.65 (1.46–1.87)	<0.001	1.31 (1.14–1.51)	<0.001

No depression at baseline assessment (*n* = 127 244)						
	Unadjusted		Age and sex adjusted		Fully adjusted^a^	
	OR (95% CI)	*p* value	OR (95% CI)	*p* value	OR (95% CI)	*p* value
Vitamin D	0.991 (0.989–0.992)^b^	<0.001	0.993 (0.991–0.994)^b^	<0.001	0.996 (0.994–0.997)^b^	<0.001
Sufficient	Reference		Reference		Reference	
Insufficient	1.30 (1.23–1.38)	<0.001	1.24 (1.16–1.31)	<0.001	1.13 (1.06–1.20)	<0.001
Deficient	1.68 (1.54–1.82)	<0.001	1.48 (1.36–1.61)	<0.001	1.22 (1.11–1.34)	<0.001

a Covariates: age, sex, deprivation, ethnicity, BMI, smoking, weekly alcohol intake, physical activity, physical disease count and season of blood sampling.

b Odds ratio for vitamin D (nmol/L) treated as a continuous value.

People with depression at baseline demonstrated similar prospective associations between vitamin D status and depression status at follow-up. After adjusting for all relevant covariates, both vitamin D insufficiency (aOR = 1.11, 95%CI 1.00–1.23) and deficiency (aOR = 1.31, 95% CI 1.14–1.51) were associated with an increased likelihood of depression at follow-up in those who were depressed at baseline. Entering vitamin D levels into the model as a continuous variable revealed that for every unit (nmol/L) increase in vitamin D, the risk of depression at follow-up declined modestly (aOR = 0.996, 95% CI 0.994–0.999).

### Sensitivity analyses

As the alcohol intake variable had missing data that exceeded the recommended threshold of 15%, we reran the logistic regression models with this imputed variable omitted. The results were very similar to those from the fully-adjusted models, confirming the validity of including these imputed variables (see online Table S1 in the Supplementary Material).

When fully-adjusted cross-sectional associations were modelled using the US IOM recommended vitamin D thresholds, results remained similar to those produced when using the SACN thresholds (see online Table S2 in the Supplementary Material). This was also the case for prospective associations between vitamin D and depression in those with no depression at baseline. However, in those with depression at baseline, vitamin D insufficiency was no longer associated with increased risk of depression at follow-up (*p* = 0.066), but deficiency remained a predictor (aOR = 1.25, 95% CI 1.10–1.42).

Prospective associations between vitamin D levels and continuous PHQ-9 scores at follow-up were significant for both those with and without depression at baseline and showed that both vitamin D insufficiency and deficiency were associated with higher PHQ-9 scores in a dose-dependent manner (see online Table S3 in the Supplementary Material). Moreover, prospective associations between baseline vitamin D status and follow-up depression were significant in the entire sample where baseline depression was included in the model as a covariate (see online Table S4 in the Supplementary Material).

The E-values and upper confidence limits for all fully adjusted analyses are provided alongside the odds ratios for the measured confounders in online Tables S5, S6 and S7 in the Supplementary Material. E-values indicate the minimum strength of association required between an unmeasured confounder and both the exposure and outcome of interest in order to overcome the statistically significant effects reported in the current study. As all the E-values were considerably larger than the odds ratios observed for well-known risk factors for depression such as age, female gender, SES and physical morbidity, it seems implausible that there is an unmeasured confounder that would overcome the statistically significant associations we have reported in the current study.

## Discussion

This is the largest study to date to examine prospective associations between vitamin D status and depression incidence in a large cohort of middle-aged adults from the UK Biobank. We observed strong evidence that in those with no depression at baseline assessment, both vitamin D insufficiency and deficiency were associated with depression at follow-up in a dose-dependent manner. This was also the case for those who had depression at the baseline assessment. What this indicates is that vitamin D insufficiency and deficiency predate the development of new-onset depression in adults of middle-age in the UK. Moreover, vitamin D deficiency – and to a lesser extent insufficiency – may also play a role in persistent depressive symptomatology.

It is possible that lower levels of vitamin D might be a manifestation of separate underlying predisposing factors for depression. For example, physical activity is known to reduce incident depression prospectively (Schuch et al., 2018). Sunlight exposure is the primary source of vitamin D, therefore lower levels of outdoor exercise might lower mood while reducing vitamin D levels. Equally, depressive symptoms might result in lower motivation to go outdoors and thus lower levels of vitamin D. However, the prospective associations described in the current study support the notion that vitamin D deficiency and insufficiency predate the development of depression, and there are a number of potential direct biological mechanisms through which vitamin D might play a role in this development. Vitamin D has been shown to regulate the expression of important neurotrophic factors involved in the proliferation, differentiation, survival and growth of neurons (Groves & Burne, 2017), and is known to be neuroprotective, preventing oxidative damage to nervous tissue (Wrzosek et al., 2013). There is also *in vitro* evidence that vitamin D maintains extracellular concentrations of serotonin in the brain (Sabir et al., 2018), a neurotransmitter central to the development of depression. Inflammation is also known to play a causal role in depression (Dregan et al., 2019; Raison & Miller, 2011), and several randomized controlled trials have found that vitamin D can lower levels of inflammatory cytokines (Cannell, Grant, & Holick, 2014). *In vitro* work has showed that there is cross-talk between vitamin D and glucocorticoid receptors within hippocampal cells which implies some sort of interaction between vitamin D and the hypothalamic–pituitary–adrenal (HPA) axis (Obradovic, Gronemeyer, Lutz, & Rein, 2006). The HPA axis has a known role in the pathogenesis of depression (Pariante & Lightman, 2008) which suggests that vitamin D might be linked to depression via HPA axis changes. There is increasing evidence for the role of the gut microbiome in depression (Winter, Hart, Charlesworth, & Sharpley, 2018) and murine studies suggest that vitamin D deficiency might bring about changes in microbiome diversity related to mood disorders (Jin et al., 2015).

The majority of evidence for the role of vitamin D in depression has come from cross-sectional associations (Anglin et al., 2013; de Oliveira, Hirani, & Biddulph, 2018; Parker et al., 2017) which were corroborated in the current study. However, cross-sectional associations provide very little information about the temporal direction of the association. The prospective results of this study indicate that vitamin D was prospectively associated with depression, and are in agreement with other studies in patients with cardiovascular disease (May et al., 2010), established depression (Milaneschi et al., 2014) and in healthy older people over 65 years (Milaneschi et al., 2010). To the best of our knowledge, this is among the largest studies to show a prospective association between vitamin D deficiency or insufficiency and depression in middle-aged adults.

Although cross-sectional associations between vitamin D and depression are well-established and there is evidence for a longitudinal association in older adults (Milaneschi et al., 2010), and adults with cardiovascular disease (May et al., 2010) and depression (Milaneschi et al., 2014), there are several prospective studies which have reported non-significant findings (Almeida, Hankey, Yeap, Golledge, & Flicker, 2015; Chan et al., 2011; Jovanova et al., 2017; Toffanello et al., 2014). This means that the current evidence is inconclusive in terms of establishing a causal role for vitamin D in depression. However, the body of evidence is small with large amounts of variation between studies. Prospective studies differ in terms of depression measurement, length of follow-up and included covariates. It is possible some studies have too few depression cases and too few cases of vitamin D deficiency to detect associations. Almost all studies to date have assessed prospective associations in older and elderly adults which might contribute to the lack of significant longitudinal findings, as the risk factors for depression may be different in this population (Muñoz et al., 2012). Only one study to date has assessed associations across the lifespan (18–65 years) and reported a prospective relationship between vitamin D and depression (Milaneschi et al., 2014). However, this study only included adults with established depression. The current study examined middle-aged adults both with and without depression at baseline that might partially explain why we detected longitudinal effects. Future research needs to adopt a lifespan approach and assess prospective associations in both healthy and depressed adults of younger and middle-age, in order to provide more conclusive evidence for the role of vitamin D in depression.

A significant amount of mixed evidence has come from trials which have assessed the effects of vitamin D supplementation as both a preventive and therapeutic agent for depressive symptoms. A meta-analysis of six of these trials assessing the effect of oral vitamin D supplementation on depressive symptoms in those with depression or at risk of depression found no significant effect (Li et al., 2014). Similarly, Gowda, Mutowo, Smith, Wluka, and Renzaho (2015) meta-analysed nine trials and found that vitamin D supplementation did not significantly reduce depression in those with subsyndromal depressive symptoms (Gowda et al., 2015). The most recent meta-analysis included four trials looking at the effect of vitamin D on depressive symptoms in patients with clinically diagnosed major depression and showed that vitamin D supplementation successfully reduced depression ratings (Vellekkatt & Menon, 2019). A recent trial in the USA found that vitamin D supplementation did not significantly affect both incident and recurrent depression over a 5-year period (Okereke et al., 2020). However, in this study, baseline vitamin D levels were generally adequate meaning that this study showed that vitamin D supplementation does not confer universal prevention against depression. In order to assess the effects of vitamin D supplementation effectively, participants need to be deficient at baseline and then receive adequate vitamin D to achieve sufficient levels during the trial. In a 2014 meta-analysis, Spedding and coworkers analysed trials separately based on this criteria (Spedding, 2014). They found that studies without ‘biological flaws’ (i.e. studies with a vitamin D deficient sample at baseline) demonstrated significant improvement in depression with vitamin D supplementation, whereas biologically flawed studies produced inconclusive results. The results of the current observational study lend support to the notion that future trials need to focus on the impact of correcting deficiency in those with deficient, and to a lesser extent insufficient, levels of vitamin D at baseline on future depression status. While trials of this kind are required, the evidence to date provides what Lally and Gaughran describe as ‘a mild signal’ for the beneficial effect of vitamin D for mood disorders (Lally & Gaughran, 2019). Although the trial evidence is not strong enough to recommend universal supplementation for depression (Menon, Kar, Suthar, & Nebhinani, 2020), the moderate effect size seen in trials without ‘biological flaws’ and trials in the clinically depressed taken together with the results of observational studies suggests that vitamin D does play a role in depression. While existing data do not allow robust causal inferences for vitamin D in the development or persistence of depressive symptoms, our study findings endorse the notion of a direct or indirect influence of vitamin D status on mental health.

### Strengths and limitations

In addition to the large sample size, data integration in the UK Biobank from multiple sources allowed for a comprehensive definition of depression caseness at baseline. The prospective study design and the ability to adjust for a considerable amount of factors known to affect both vitamin D status and depression are further strengths of the current study.

Several limitations need consideration. A major limitation associated with observational studies is difficulty in establishing causality and residual confounding. Although we controlled for a significant number of relevant confounders, we cannot reject the possibility of unmeasured confounders that might bias our results. For example, although we adjusted for a comprehensive number of physical conditions in the current study, it is possible that vitamin D might be a marker of unmeasured chronic non-specific disease, rather than a factor relating specifically to depression (Jovanova et al., 2017). We were also unable to consider levels of parathyroid hormone, which is thought to play a role in the link between vitamin D and depression (May et al., 2010). However, the E-value methodology (VanderWeele & Ding, 2017) which assesses the robustness of results to unmeasured confounding showed that it was implausible that there might be any one unmeasured confounder that would overcome the statistically significant associations we report in this study.

Vitamin D was only assessed at one time point meaning that measuring changes in vitamin D over time, which would have allowed for a more robust assessment of causal association with depression, was not possible. However, vitamin D status has been shown to be relatively stable over time (McKibben et al., 2016; van Schoor et al., 2014), and there is prospective evidence to suggest that single vitamin D measures can be used to predict future health outcomes (Jorde et al., 2010). Although a comprehensive measure of depression was used at baseline, depression at follow-up was measured using the PHQ-9. This inconsistency between measurement of depression across time points might introduce some discrepancies in depression caseness at baseline and follow-up. However, the baseline depression measurement included the PHQ-2 which shows very similar sensitivity for diagnosis of depression to the PHQ-9 (Arroll et al., 2010). Moreover, it is worth bearing in mind that both the PHQ-2 and PHQ-9 are not measures of clinical depression, although they have shown suitable psychometric properties when compared to other longer clinician-administered depression instruments (Gilbody, Richards, Brealey, & Hewitt, 2007). The UK Biobank comprises middle-aged participants which is one of the strengths of the current study. However, the generalizability of the results is limited to this population subgroup. The UK Biobank cohort is also known to differ from the general UK population in terms of demographic (more female, less deprived) and health (less smoking, lower alcohol intake, fewer self-reported health conditions) factors which will also affect the generalizability of results (Fry et al., 2017). However, more comprehensive UK data may have elicited stronger associations between vitamin D and depression seeing as rates of depression and vitamin D deficiency/insufficiency might have been higher in a more representative sample.

## Conclusion

This is the largest population-based study to show prospective associations between vitamin D status and depression in middle-aged adults. The results suggest that both vitamin D deficiency and insufficiency may help discriminate between adults at increased risk of subsequent depression, as well as act as biomarkers for persistent depressive symptoms in those who are already depressed.

## Supplementary material

For supplementary material accompanying this paper visit https://doi.org/10.1017/S0033291720003657. click here to view supplementary material
