# Supplementary material for: Prospective associations between vitamin D and depression in middle-aged adults: findings from the UK Biobank cohort
Source: Psychol Med. 2020 Oct 21;52(10):1866–74. doi: 10.1017/S0033291720003657 (PMC9340850; doi:10.1017/S0033291720003657)
Supplement: Supplementary file 1 [file S0033291720003657sup001.docx]

**Supplementary Material**

(1) As the alcohol intake variable had missing data that exceeded the recommended threshold of 15%, we reran the models with the imputed alcohol variable omitted:

| **Table S1.** Associations between vitamin D and depression with alcohol intake omitted from the regression models | | | | | | |
| --- | --- | --- | --- | --- | --- | --- |
|  | **Cross-sectional** | | **Prospective (no depression at baseline)** | | **Prospective (depression at baseline)** | |
|  | *OR (95% CI)* | *p value* | *OR (95% CI)* | *p value* | *OR (95% CI)* | *p value* |
| Vitamin D | 0.995 (0.994-0.996)† | <.001 | 0.996 (0.994-0.997)† | <.001 | 0.996 (0.994-0.999)† | 0.002 |
| *Sufficient* | Reference |  | Reference |  | Reference |  |
| *Insufficient* | 1.15 (1.10-1.20) | <.001 | 1.13 (1.06-1.20) | <.001 | 1.11 (1.00-1.23) | 0.042 |
| *Deficient* | 1.34 (1.26-1.43) | <.001 | 1.22 (1.11-1.34) | <.001 | 1.32 (1.15-1.52) | <.001 |
| Covariates: Age, sex, deprivation, ethnicity, BMI, smoking, physical activity, physical disease count, and season of blood sampling  † Odds ratio for Vitamin D (nmol/L) treated as a continuous value | | | | | | |

(2) Analyses were rerun using the US IOM vitamin D thresholds for comparative purposes:

| **Table S2.** Associations between vitamin D levels and depression at follow-up (PHQ-9) using the US Institute of Medicine (IOM) thresholds | | | | | | |
| --- | --- | --- | --- | --- | --- | --- |
| Cross-sectional associations | | | | | | |
|  | **Unadjusted** | | **Age and sex adjusted** | | **Fully adjusted*** | |
|  | *OR (95% CI)* | *p value* | *OR (95% CI)* | *p value* | *OR (95% CI)* | *p value* |
| *Sufficient* | Reference |  | Reference |  | Reference |  |
| *Insufficient* | 1.22 (1.17-1.28) | <.001 | 1.19 (1.14-1.24) | <.001 | 1.14 (1.09-1.20) | <.001 |
| *Deficient* | 1.51 (1.44-1.59) | <.001 | 1.42 (1.35-1.49) | <.001 | 1.29 (1.22-1.36) | <.001 |
| Prospective associations in those with depression at baseline assessment (n=11,884) | | | | | | |
|  | **Unadjusted** | | **Age and sex adjusted** | | **Fully adjusted*** | |
|  | *OR (95% CI)* | *p value* | *OR (95% CI)* | *p value* | *OR (95% CI)* | *p value* |
| *Sufficient* | Reference |  | Reference |  | Reference |  |
| *Insufficient* | 1.27 (1.15-1.41) | <.001 | 1.22 (1.10-1.35) | <.001 | 1.11 (0.99-1.23) | 0.066 |
| *Deficient* | 1.66 (1.49-1.85) | <.001 | 1.54 (1.38-1.71) | <.001 | 1.25 (1.10-1.42) | <.001 |
| Prospective associations in those with no depression at baseline assessment (n=127,244) | | | | | | |
|  | **Unadjusted** | | **Age and sex adjusted** | | **Fully adjusted*** | |
|  | *OR (95% CI)* | *p value* | *OR (95% CI)* | *p value* | *OR (95% CI)* | *p value* |
| *Sufficient* | Reference |  | Reference |  | Reference |  |
| *Insufficient* | 1.26 (1.19-1.35) | <.001 | 1.21 (1.13-1.29) | <.001 | 1.11 (1.04-1.19) | 0.002 |
| *Deficient* | 1.60 (1.49-1.72) | <.001 | 1.44 (1.34-1.55) | <.001 | 1.22 (1.13-1.32) | <.001 |
| *Covariates: Age, sex, deprivation, ethnicity, BMI, smoking, weekly alcohol intake, physical activity, physical disease count, and season of blood sampling. | | | | | | |

(3) Analyses were performed to examine the impact of vitamin D status on continuous PHQ-9 scores, as a binary PHQ-9 depression outcome variable was used in the main analyses:

| **Table S3.** Prospective associations between vitamin D levels at baseline and continuous PHQ-9 scores at follow-up | | | | | | |
| --- | --- | --- | --- | --- | --- | --- |
| Prospective associations in those with depression at baseline assessment (n=11,884) | | | | | | |
|  | **Unadjusted** | | **Age and sex adjusted** | | **Fully adjusted*** | |
|  | *β* | *p value* | *β* | *p value* | *β* | *p value* |
| *Sufficient* | Reference |  | Reference |  | Reference |  |
| *Insufficient* | 0.78 | <.001 | 0.62 | <.001 | 0.27 | 0.022 |
| *Deficient* | 1.70 | <.001 | 1.41 | <.001 | 0.60 | <.001 |
| Prospective associations in those with no depression at baseline assessment (n=127,244) | | | | | | |
|  | **Unadjusted** | | **Age and sex adjusted** | | **Fully adjusted*** | |
|  | *β* | *p value* | *β* | *p value* | *β* | *p value* |
| *Sufficient* | Reference |  | Reference |  | Reference |  |
| *Insufficient* | 0.29 | <.001 | 0.23 | <.001 | 0.14 | <.001 |
| *Deficient* | 0.68 | <.001 | 0.54 | <.001 | 0.36 | <.001 |
| *Covariates: Age, sex, deprivation, ethnicity, BMI, smoking, weekly alcohol intake, physical activity, physical disease count, and season of blood sampling. | | | | | | |

(4) We examined associations between baseline vitamin D status and depression at follow-up in the entire sample, including depression at baseline in the model as a covariate:

| **Table S4.** Fully-adjusted* associations between baseline vitamin D status and depression at follow-up (PHQ-9) in the entire sample (n=139,128) | | | | | |  |
| --- | --- | --- | --- | --- | --- | --- |
|  | **Sufficient** | **Insufficient** | | **Deficient** | |  |
|  |  | *OR (95% CI)* | *p value* | *OR (95% CI)* | *p value* |  |
| Baseline depression | Reference | 1.12 (1.06-1.19) | <0.001 | 1.25 (1.16-1.35) | <0.001 |  |
| *Covariates: Age, sex, deprivation, ethnicity, BMI, smoking, weekly alcohol intake, physical activity, physical disease count, season of blood sampling, and baseline depression status. | | | | | |  |

(5) The E-value methodology (VanderWeele & Ding, 2017) was used to assess the potential effect of unmeasured confounding. This method estimates the minimum strength of the association that would be required between an unmeasured confounder and both vitamin D status and depression to overcome the statistically significant effects from fully-adjusted models reported in the current study.

For the cross-sectional analyses, the E-value for vitamin D insufficiency and deficiency were 1.59 (upper CI limit: 1.46) and 2.01 (upper CI limit: 1.81) respectively. This means that an unmeasured confounder would need to be associated with both vitamin D and depression at baseline by an odds ratio of 1.59 for insufficient and 2.01 for deficient vitamin D levels in order to overcome the statistically significant associations we have reported in the current study. These odds ratios are above and beyond the measured confounders included in the model which are listed in Table S5:

| **Table S5.** Fully-adjusted cross-sectional associations between vitamin D and depression caseness at baseline with E-values | | | | |
| --- | --- | --- | --- | --- |
|  | *OR (95% CI)* | *p value* | *E-value* | *Upper CI limit* |
| Vitamin D |  |  |  |  |
| *Sufficient* | Reference |  |  |  |
| *Insufficient* | 1.16 (1.11 – 1.21) | <0.001 | 1.59 | 1.46 |
| *Deficient* | 1.34 (1.25 – 1.42) | <0.001 | 2.01 | 1.81 |
| Age | 0.96 (0.96 – 0.97) | <0.001 |  |  |
| Sex |  |  |  |  |
| *Female* | Reference |  |  |  |
| *Male* | 0.62 (0.59 – 0.65) | <0.001 |  |  |
| Ethnicity |  |  |  |  |
| *White* | Reference |  |  |  |
| *Non-white* | 1.14 (1.03 – 1.26) | 0.013 |  |  |
| BMI *(kg/m^2^)* | 1.02 (1.02- 1.26) | <0.001 |  |  |
| Deprivation | 1.04 (1.03 – 1.04) | <0.001 |  |  |
| Smoking status |  |  |  |  |
| *Never smoked* | Reference |  |  |  |
| *Past/current smoker* | 1.15 (1.11 – 1.18) | <0.001 |  |  |
| Alcohol intake *(units per week*) | 1.01 (1.01 – 1.01) | <0.001 |  |  |
| Physical activity |  |  |  |  |
| *None* | Reference |  |  |  |
| *Low* | 0.62 (0.54 – 0.72) | <0.001 |  |  |
| *Moderate* | 0.56 (-0.49 – 0.65) | <0.001 |  |  |
| *Vigorous* | 0.57 (0.49 – 0.65) | <0.001 |  |  |
| Physical disease count | 1.33 (1.31 – 1.35) | <0.001 |  |  |
| Season of blood sampling |  |  |  |  |
| *Winter* | Reference |  |  |  |
| *Spring* | 1.03 (0.97 – 1.09) | 0.307 |  |  |
| *Summer* | 1.15 (1.08 – 1.22) | <0.001 |  |  |
| *Autumn* | 1.04 (0.98 – 1.10) | 0.245 |  |  |

For the prospective analyses in those with depression at baseline, the E-values for vitamin D insufficiency and deficiency were 1.46 (upper CI limit: 1.00) and 1.95 (upper CI limit: 1.54) respectively. This means that an unmeasured confounder would need to be associated with both vitamin D and depression at follow-up in those with depression at baseline by an odds ratio of 1.46 for insufficient and 1.95 for deficient vitamin D levels in order to overcome the statistically significant associations we have reported in the current study. These odds ratios are considerably higher than those of the measured confounders included in the model listed in Table S6:

| **Table S6.** Fully-adjusted prospective associations between vitamin D and depression (PHQ-9) at follow-up in participants with depression at baseline with E-values | | | | |
| --- | --- | --- | --- | --- |
|  | *OR (95% CI)* | *p value* | *E-value* | *Upper CI limit* |
| Vitamin D |  |  |  |  |
| *Sufficient* | Reference |  |  |  |
| *Insufficient* | 1.11 (1.00 – 1.23) | 0.042 | 1.46 | 1.00 |
| *Deficient* | 1.31 (1.14 – 1.51) | <0.001 | 1.95 | 1.54 |
| Age | 0.95 (0.95 – 0.96) | <0.001 |  |  |
| Sex |  |  |  |  |
| *Female* | Reference |  |  |  |
| *Male* | 0.96 (0.87 – 1.06) | 0.451 |  |  |
| Ethnicity |  |  |  |  |
| *White* | Reference |  |  |  |
| *Non-white* | 0.85 (0.68 – 1.07) | 0.173 |  |  |
| BMI *(kg/m^2^)* | 1.04 (1.03 – 1.05) | <0.001 |  |  |
| Deprivation | 1.06 (1.04 – 1.07) | <0.001 |  |  |
| Smoking status |  |  |  |  |
| *Never smoked* | Reference |  |  |  |
| *Past/current smoker* | 1.06 (0.97 – 1.16) | 0.220 |  |  |
| Alcohol intake *(units per week*) | 1.01 (1.00 – 1.01) | <0.001 |  |  |
| Physical activity |  |  |  |  |
| *None* | Reference |  |  |  |
| *Low* | 0.53 (0.40 – 0.69) | <0.001 |  |  |
| *Moderate* | 0.43 (0.33 – 0.56) | <0.001 |  |  |
| *Vigorous* | 0.40 (0.31 – 0.52) | <0.001 |  |  |
| Physical disease count | 1.22 (1.17 – 1.26) | <0.001 |  |  |
| Season of blood sampling |  |  |  |  |
| *Winter* | Reference |  |  |  |
| *Spring* | 1.05 (0.92 – 1.20) | 0.451 |  |  |
| *Summer* | 1.14 (0.99 – 1.30) | 0.066 |  |  |
| *Autumn* | 1.04 (0.91 – 1.20) | 0.548 |  |  |

For the prospective analyses in those without depression at baseline, the E-value for vitamin D insufficiency and deficiency were 1.51 (upper CI limit: 1.31) and 1.74 (upper CI limit: 1.46) respectively. This means that an unmeasured confounder would need to be associated with both vitamin D and depression at follow-up in those without depression at baseline by an odds ratio of 1.51 for insufficient and 1.74 for deficient vitamin D levels in order to overcome the statistically significant associations we have reported in the current study. These odds ratios are considerably higher than those of the measured confounders included in the model listed in Table S7:

| **Table S7.** Fully-adjusted prospective associations between vitamin D and depression (PHQ-9) at follow-up in participants with no depression at baseline, with E-values | | | | |
| --- | --- | --- | --- | --- |
|  | *OR (95% CI)* | *p value* | *E-value* | *Upper CI limit* |
| Vitamin D |  |  |  |  |
| *Sufficient* | Reference |  |  |  |
| *Insufficient* | 1.13 (1.06 – 1.20) | <0.001 | 1.51 | 1.31 |
| *Deficient* | 1.22 (1.11 – 1.34) | <0.001 | 1.74 | 1.46 |
| Age | 0.94 (0.93 – 0.94) | <0.001 |  |  |
| Sex |  |  |  |  |
| *Female* | Reference |  |  |  |
| *Male* | 0.70 (0.66 – 0.75) | <0.001 |  |  |
| Ethnicity |  |  |  |  |
| *White* | Reference |  |  |  |
| *Non-white* | 1.02 (0.87 – 1.18) | 0.842 |  |  |
| BMI *(kg/m^2^)* | 1.05 (1.04 – 1.05) | <0.001 |  |  |
| Deprivation | 1.06 (1.04 – 1.07) | <0.001 |  |  |
| Smoking status |  |  |  |  |
| *Never smoked* | Reference |  |  |  |
| *Past/current smoker* | 1.30 (1.22 – 1.37) | <0.001 |  |  |
| Alcohol intake *(units per week*) | 1.01 (1.00 – 1.01) | <0.001 |  |  |
| Physical activity |  |  |  |  |
| *None* | Reference |  |  |  |
| *Low* | 0.55 (-.45 – 0.67) | <0.001 |  |  |
| *Moderate* | 0.49 (0.41 – 0.59) | <0.001 |  |  |
| *Vigorous* | 0.47 90.39 – 0.57) | <0.001 |  |  |
| Physical disease count | 1.38 (1.34 – 1.41) | <0.001 |  |  |
| Season of blood sampling |  |  |  |  |
| *Winter* | Reference |  |  |  |
| *Spring* | 1.03 (0.95 – 1.12) | 0.481 |  |  |
| *Summer* | 1.12 (1.02 – 1.22) | 0.014 |  |  |
| *Autumn* | 1.09 (1.00 – 1.19) | 0.044 |  |  |

It seems implausible that there is an unmeasured confounder that would overcome the statistical significance reported in the current study, given that the E-values were much larger than the observed odds ratios for well-known risk factors for depression such as age, female gender, low socioeconomic status, and physical morbidity.
